# Adiponectin/resistin interplay in serum and in adipose tissue of obese and normal-weight individuals

**DOI:** 10.1186/s13098-017-0293-2

**Published:** 2017-12-01

**Authors:** Marta Izabela Jonas, Alina Kurylowicz, Zbigniew Bartoszewicz, Wojciech Lisik, Maurycy Jonas, Justyna Domienik-Karlowicz, Monika Puzianowska-Kuznicka

**Affiliations:** 1 Department of Human Epigenetics, Mossakowski Medical Research Centre, Polish Academy of Sciences, 5 Pawinskiego Street, 02-106 Warsaw, Poland; 2 Department of General and Transplantation Surgery, Medical University of Warsaw, 59 Nowogrodzka Street, 02-005 Warsaw, Poland; 3 Department of Internal Medicine and Cardiology, Medical University of Warsaw, 5 Lindleya Street, 02-005 Warsaw, Poland; 4 Department of Geriatrics and Gerontology, Medical Centre of Postgraduate Education, 99 Marymoncka Street, 01-813 Warsaw, Poland

**Keywords:** Adiponectin, Resistin, Adiponectin/resistin index, Obesity, Serum, Adipose tissue

## Abstract

**Background:**

The interplay between adiponectin and resistin, the two adipokines of opposite effects, may determine the metabolic profile of obese individuals and development of obesity-related complications. The current study was conducted to assess how adiponectin/resistin interplay in sera and adipose tissues may influence the metabolic profile of obese and normal-weight subjects.

**Methods:**

Concentrations of adiponectin and resistin were measured on protein level by immunoassay in visceral and subcutaneous adipose tissues from 50 obese (body mass index > 40 kg/m^2^) and 28 normal-weight (body mass index 20–24.9 kg/m^2^) individuals. Simultaneously expression of *ADIPOQ* and *RETN* (encoding adiponectin and resistin, respectively) was assessed on mRNA level by real-time PCR.

**Results:**

*ADIPOQ* mRNA (P = 0.0001) and adiponectin protein (P = 0.0013) levels were lower, while *RETN* mRNA (P = 0.0338) and resistin (P < 0.0001)—higher in subcutaneous adipose tissues of obese subjects. *ADIPOQ* and *RETN* mRNA levels did not correlate with protein concentrations in the investigated adipose tissues. In obesity adiponectin serum concentrations correlated positively with *ADIPOQ* mRNA in subcutaneous adipose tissue (P = 0.005) and negatively with protein levels in visceral adipose tissue (P = 0.001). Obesity was associated with higher adiponectin–resistin index value in sera (P < 0.0001) and decreased in subcutaneous adipose tissue (P < 0.001), but only adiponectin–resistin index measured in sera was significantly higher in obese with the metabolic syndrome (P = 0.04).

**Conclusions:**

Obesity affects synthesis of adiponectin and resistin mainly in subcutaneous adipose tissue. The adiponectin–resistin index assessed in the adipose tissues has a different prognostic value compared to the adiponectin–resistin index in serum and does not reflect a metabolic risk in obese individuals.

## Background

Identification of adipokines widened our understanding of the role of adipose tissue which is now considered not only an energy storage, but also an important endocrine organ. Characterization of adipokines activities allowed to establish the link between excess adiposity and development of obesity-related complications [1].

Adiponectin is a protein hormone almost exclusively produced in adipocytes, that has many favorable metabolic properties, e.g. acts as anti-inflammatory, anti-atherogenic and anti-oxidative factor [2]. Adiponectin exhibits insulin-sensitizing effect and may increase insulin secretion [3]. Acting centrally, adiponectin increases energy expenditure and, therefore, prevents from excessive energy accumulation [4]. In several studies adiponectin levels measured in serum of obese individuals were significantly lower compared to the normal-weight subjects and correlated negatively with the presence of obesity-related complications [5–7].

Resistin is a peptide with biological properties opposite to adiponectin. It is expressed mainly in the adipose tissue, but it was found also in other tissues [8]. Notably, in isolated human adipocytes, resistin expression is very low, and its content in adipose tissue is proportional to the intensity of macrophages infiltration, which are the main source of this adipokine [9, 10]. Obesity in humans was found to be associated with high resistin serum levels, this view however is not unanimous [11–13]. High serum resistin level, due to its pro-inflammatory properties, was linked to the development of insulin resistance and type 2 diabetes (T2DM), to atherosclerosis and cardiovascular diseases in rodents and in some human studies [13, 14].

Taking into account the opposite effects of adiponectin and resistin, it was suggested that the interplay between these two adipokines may determine the metabolic profile of obese individuals and, possibly, be responsible for the development of obesity-related complications. Adiponectin–resistin (AR) index was proposed as an indicator of metabolic risk in obesity [15]. Subsequently, AR was found to positively correlate with risk of development of T2DM and the metabolic syndrome (MS).

Obesity leads to the changes in metabolic and secretory activity of various adipose tissue depots [16, 17]. It is not known however which adipose tissue depot, visceral (VAT) or subcutaneous (SAT) plays a dominant role in the synthesis and secretion of adiponectin and resistin in health and in obesity, because analyses of the *ADIPQ* and *RETN* expression (encoding adiponectin and resistin, respectively) in human adipose tissue were performed on the mRNA level [18–20].

## Methods

### Aim of the study

The aim of the study was to assess how obesity influences the adiponectin/resistin interplay in serum and expression of the *ADIPQ* and *RETN* genes and proteins in various adipose tissue depots and to establish whether these values correlate with clinical characteristics of obese individuals.

### Study subjects and tissues

The group of obese study participants consisted of 50 individuals (43 females and 7 males) aged 20–59 years (mean 42.02 years, standard deviation (SD) ± 10.33 years). According to the World Health Organization classification, all patients were diagnosed with class III obesity and their mean body mass index (BMI) was 47.02 kg/m^2^ (SD ± 4.91 kg/m^2^, range 35.43–59.26 kg/m^2^), while mean waist circumference was 1.25 m (SD ± 0.18 m, range 0.97–1.67 m). Since weight of some obese study participants exceeded 150 kg, dual-energy X-ray absorptiometry (DXA) or magnetic resonance (MRI)-based methods could not be used to assess their body composition on the available equipment. Therefore, for this purpose we used the Segmental Body Composition Analyser BC-418 MA eight-contact electrode system (Tanita Corp., Tokyo, Japan) based on the bioelectric impedance method. The mean adipose tissue content (assessed by predictive equation described by Jebb et al. and by Franssen et al. [21, 22]) was 63.86% (SD ± 8.74%, range 47.9–79.4%). Twenty-five obese study participants (50%) were diagnosed with T2DM or prediabetes (impaired fasting glucose or impaired glucose tolerance), 31 (62%) with hypertension and 30 (60%) with hyperlipidemia. Based on the International Diabetes Federation (IDF, http://www.idf.com) criteria for the Europeans, the metabolic syndrome (MS) was diagnosed in 33 (66%) of the obese individuals.

Control group consisted of 28 subjects (20 females, 8 males), aged 22–63 years (mean 46.46 years, SD ± 14.6 years), whose BMI was within normal range (20.1–24.93 kg/m^2^, mean 23.28 ± 1.65 kg/m^2^). Although the body composition was not assessed in this group, based on the normal blood test results and negative medical history of chronic diseases including components of MS (as described previously [17]) they were considered metabolically healthy.

All surgical procedures were performed in the Department of General and Transplantation Surgery, Medical University of Warsaw. Before surgery, 15 ml of venous blood was collected from all study participants to obtain serum for biochemical and immunological measurements. Fifty pairs of visceral and subcutaneous adipose tissues were collected from obese individuals during bariatric surgery. Twenty-eight pairs of control adipose tissue samples were obtained from normal-weight individuals during elective cholecystectomy. After collection, all 156 adipose tissue samples were frozen at − 80 °C and subsequently homogenized in liquid nitrogen.

### Measurement of adiponectin, resistin and total protein concentrations

Isolation of protein fraction from adipose tissues was performed as described previously [17]. Adiponectin monomers and resistin concentrations in serum and in protein extracts from adipose tissues were assessed with ELISA-based chemiluminescent custom-made Q-plex Custom array (Quansys Bioscience, West Logan, UT, USA). Luminescence was measured in the Molecular Imager Versa Doc™ MP 5000 System (Bio-Rad, Hercules, CA, USA) according to the manufacturer’s guidelines. Results were analyzed with the Q-View software version 2.17 (Quansys Bioscience, West Logan, UT, USA).

Adiponectin and resistin levels in adipose tissues were normalized to total protein concentration in these tissues established with the Pierce BCA Protein Assay Kit (Thermo Fisher Scientific, Rockford, IL, USA). The mean protein concentrations in extracts from VAT and SAT samples obtained from obese (O) and normal-weight (N) individuals were not significantly different (P > 0.05).

Adiponectin–resistin (AR) index was calculated by the formula proposed by Lau and Muniandy [15]: AR index = 1 + log_10_ (R_0_) − log_10_ (A_0_). The lower the concentration of adiponectin and the higher the concentration of resistin, the higher the value of AR.

### Isolation of total RNA, reverse transcription and analysis of ADIPQ and RETN expression by real-time PCR

Previously described methods [17] were used for total RNA isolation and for reverse transcription of total RNA to cDNA. Real-time PCRs were performed in triplicate in LightCycler 480 Instrument II (Roche, Mannheim, Germany), as described previously [17]. Specific primers and real-time PCR conditions used for the analysis of *ADIPQ* or *RETN* are enlisted in Table 1.

**Table 1:** Primers used for the analysis of adiponectin and resistin expression on mRNA level in adipose tissues

Gene	Gene description	Gene bank	Primers		Annealing (^o^C)
*ADIPOQ*	Adiponectin	NM_001177800.1	F	5′GGTCTCGAACTCCTGGCCTA3′	60
			R	5′TGAGATATCGACTGGGCATGGT3′	
*RETN*	Resisitin	NM_020415.3	F	5′GCTGTTGGTGTCTAGCAAGAC3′	59
			R	5′CATCATCATCATCATCTCCAG3′	
*ACTB*	β-Actin	NM_001101	F	5′CAGCCTGGATAGCAACG-TACA3′	61
			R	5′TTCTACAATGAGCTGCGTGTG3′	

### Statistical analysis

Differences in (i) *ADIPQ* and *RETN* mRNA, and in (ii) adiponectin and resistin protein levels between the studied tissues/sera, as well as between genders were assessed with the Statistica software package v.10 (StatSoft, Tulsa, OK) using the Student’s t/Mann–Whitney U test or Kruskal–Wallis analysis of variance. All correlations between quantitative values were analyzed with the Spearman correlation test and the Spearman’s correlation coefficient value r_s_ was calculated. Normality of distribution and homogeneity of the variance were assessed with the Shapiro–Wilk and Levene’s tests, respectively.

## Results

### Adiponectin and resistin concentrations in serum from obese and normal-weight individuals

The initial analysis revealed no significant gender-related differences in serum adiponectin and resistin levels in the studied groups; therefore, all further analyses were performed without stratification for gender.

**Fig. 1:**
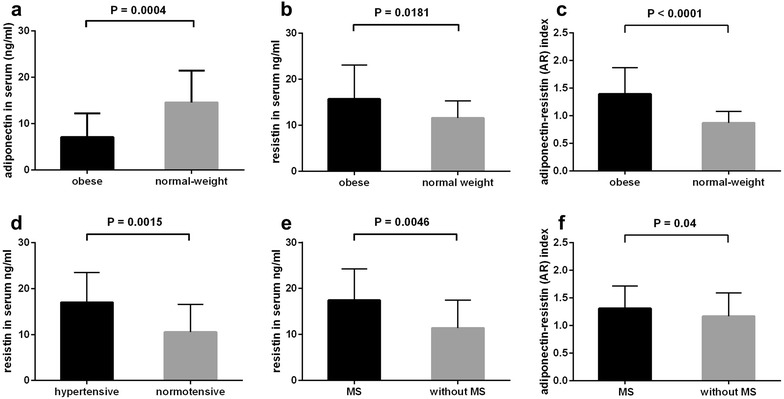
Adiponectin (**a**), resistin (**b**) and adiponectin–resistin (AR) index (**c**) in sera of obese and normal-weight individuals. Comparison of resistin serum levels in individuals stratified according to presence of hypertension (**d**) and metabolic syndrome (MS, **e**). Comparison of adiponectin–resistin (AR) index in obese individuals stratified according to presence of MS (**f**). Results are presented as mean ± standard deviation

The mean serum adiponectin level was significantly lower in the obese individuals compared to the normal-weight study participants (7.06 ng/ml vs 14.57 ng/ml, P = 0.0004, Fig. 1a), and the difference concerned both “healthy” obese (P = 0.0004) and these who suffered from at least one obesity-related complication (P < 0.0001). The mean serum adiponectin levels did not differ between the “healthy” and “unhealthy” obese subjects. There were no significant correlations between serum adiponectin and glucose, C-reactive protein (CRP) and lipid profile in both obese sub-groups.

In contrast, the mean serum resistin concentration was higher in the obese subjects (15.69 ng/ml vs 11.56 ng/ml, P = 0.018, Fig. 1b) and positively correlated with serum triglycerides (r_s_ = 0.399, P = 0.04) and negatively with high density lipoprotein (HDL) level (r_s_ = − 0.475, P = 0.016). Moreover, when the obese study participants were stratified according to the presence of MS and its components (hypertension, diabetes and hyperlipidemia), the mean serum resistin levels were higher in hypertensive patients (P = 0.0015, Fig. 1d) and in these who met the MS criteria (P = 0.0046, Fig. 1e) than in these who were normotensive and did not have MS, respectively. In the “healthy” obese subjects, the mean serum resistin concentrations were not significantly different than in the normal-weight individuals.

The mean adiponectin–resistin (AR) index was significantly higher in serum of obese subjects compared to serum of normal-weight individuals (P < 0.0001) (Fig. 1c). The mean AR index value was also higher in the obese study participants who met the MS criteria than in these who did not (P = 0.04, Fig. 1f). Notably, the mean AR index value in “healthy” obese subjects was similar to that in the normal-weight individuals.

### Expression of adiponectin and resistin in adipose tissues from obese and normal-weight individuals

Next, the expression of *ADIPOQ* and *RETN* was measured on the mRNA and protein levels in visceral and subcutaneous adipose tissue samples collected from the obese and normal-weight study participants. Since the mean *ADIPOQ* and *RETN* mRNA levels, as well as adiponectin and resistin protein concentrations did not differ in the adipose tissue of males and females, all subsequent analyses were performed for all study participants together.

**Fig. 2:**
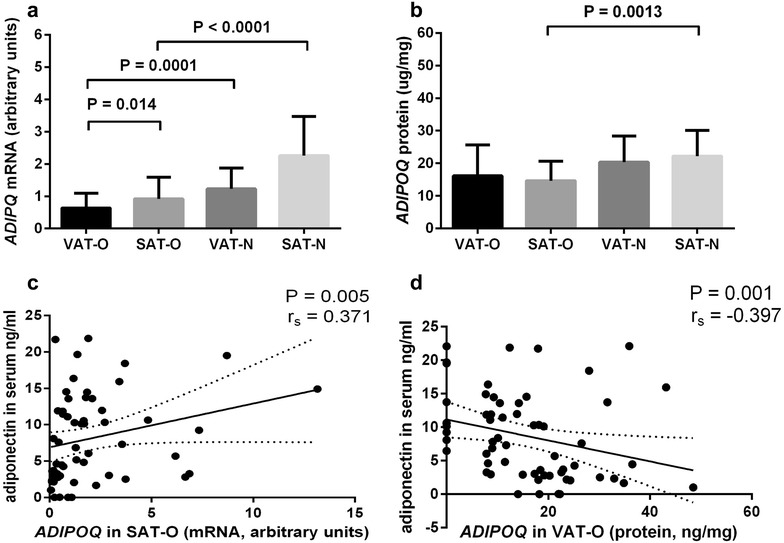
The mean *ADIPOQ* mRNA levels (**a**) and mean adiponectin protein concentrations (**b**) in the visceral (VAT) and subcutaneous (SAT) adipose tissues of obese (O) and normal-weight (N) individuals. Results are shown as mean ± standard deviation. Correlation of adiponectin serum concentrations with *ADIPOQ* mRNA levels in SAT (**c**) and with adiponectin protein concentration in VAT (**d**) of obese individuals

The mean *ADIPOQ* mRNA level (Fig. 2a) was lower in adipose tissues of obese study participants than of normal-weight study subjects and the difference concerned both VAT (P = 0.0001) and SAT (P < 0.0001). However, adiponectin protein concentrations (Fig. 2b) were lower only in SAT of obese study participants compared to SAT of normal-weight individuals (P = 0.0013). Additionally, obesity was associated with higher *ADIPOQ* mRNA level in SAT compared to VAT (P = 0.014). No significant correlation was observed between the *ADIPOQ* mRNA and adiponectin protein levels in the investigated tissues. No significant differences in adiponectin protein concentrations were observed between the “healthy” obese subjects and these with obesity-related complications.

In obese individuals adiponectin serum concentrations correlated positively with *ADIPOQ* mRNA in SAT (P = 0.005, r_s_ = 0.371, Fig. 2c) and negatively with adiponectin protein levels in VAT (P = 0.001, r_s_ = − 0.397, Fig. 2d). In normal-weight subjects no significant correlations between the adiponectin serum levels and *ADIPOQ* expression either on mRNA or on protein level were observed.

**Fig. 3:**
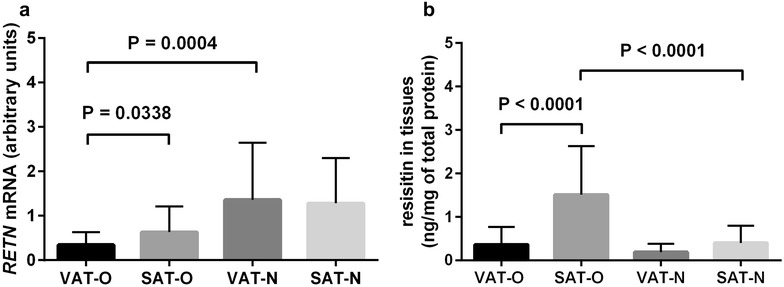
The mean *RETN* mRNA levels (**a**) and mean resistin protein concentrations (**b**) in the visceral (VAT) and subcutaneous (SAT) adipose tissues of obese (O) and normal-weight (N) individuals. Results are shown as mean ± standard deviation

Both mean *RETN* mRNA and resistin protein levels were higher in SAT of obese subjects compared to SAT of the control group (P = 0.0338 and P < 0.0001, respectively, Fig. 3a, b). While the mean *RETN* mRNA was significantly lower in VAT of obese study participants than in VAT of normal-weight subjects (P = 0.0004, Fig. 3a), the mean resistin protein concentration was higher in SAT of obese subjects than in SAT of normal-weight individuals (P < 0.0001, Fig. 3b). In addition, *RETN* expression assessed at the mRNA level did not correlate with resistin protein concentrations in the same tissues. Notably, the mean resistin protein level was higher in obese individuals who suffered from obesity-related complications than in “healthy” obese subjects both in VAT (P = 0.005) and SAT (P = 0.017). Resistin serum levels correlated neither with *RETN* mRNA nor with resistin protein concentrations in the investigated tissues.

Adiponectin–resistin index assessment in tissues showed that obesity was associated with a decreased AR value in SAT (P < 0.001) as compared to normal-weight individuals, while in VAT we found no difference in AR value between the studied groups. The AR value in VAT was higher than in SAT of obese individuals (P = 0.0021), while in the normal-weight subjects no differences were observed. The AR index value in tissues did not differ between the “healthy” and “unhealthy” obese study participants.

### Correlation of adiponectin and resistin protein levels in adipose tissues with clinical and biochemical parameters

**Fig. 4:**
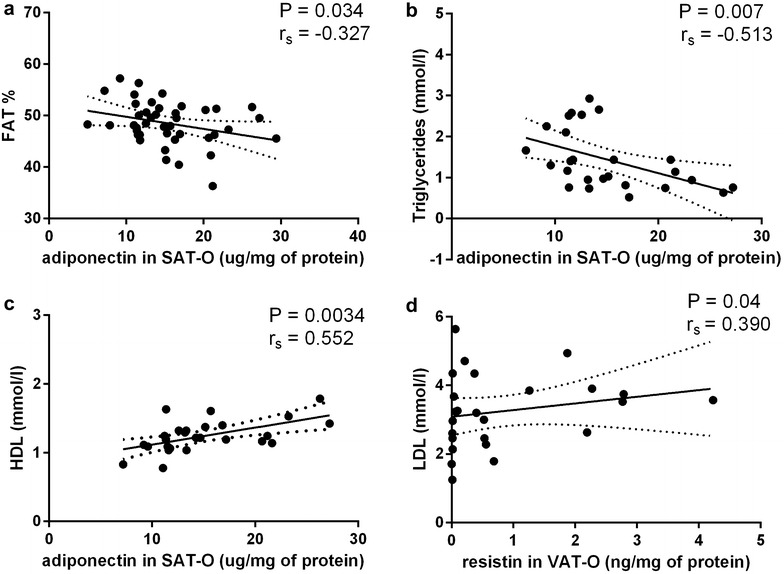
Correlation of adiponectin protein level in subcutaneous adipose tissue (SAT) of obese individuals (O) with adipose tissue content (**a**), serum triglycerides (**b**) and high density lipoproteins (HDL) serum levels (**c**). Correlation of resistin protein level in SAT-O with low density lipoproteins (LDL) serum levels (**d**)

Assessment of adipose tissue adiponectin and resistin protein levels correlation with basic clinical (BMI, adipose tissue content, waist circumference) and serum biochemical (glucose, CRP, lipid profile) parameters showed that in the obese study participants adiponectin concentrations in SAT correlated negatively with adipose tissue content (r_s_ = − 0.327, P = 0.034, Fig. 4a) and serum triglycerides (r_s_ = − 0.513, P = 0.007, Fig. 4b), but positively with HDL serum levels (r_s_ = 0.552, P = 0.0034, Fig. 4c). In turn, resistin level in SAT correlated positively with LDL serum concentration (r_s_ = 0.390, P = 0.04, Fig. 4d). No significant correlations between adiponectin and/or resistin levels in VAT and clinical and biochemical parameters were observed.

## Discussion

In this study we investigated how adiponectin/resistin interplay in serum and in various adipose tissue depots may affect metabolic profile of the obese and normal-weight individuals. To do this, we were the first to measure adiponectin and resistin protein levels directly in adipose tissues using immunoassay method. We found no correlations between adiponectin and resistin levels in VAT or in SAT with their concentrations in serum. In addition, we found a negative association between obesity and the serum AR index value but not with adipose tissue AR. We also observed a number of correlations between different metabolic parameters and adiponectin and resistin concentrations in serum and/or in adipose tissue compartments in obese individuals.

Our results regarding lower adiponectin serum level in the obese than in normal-weight subjects are consistent with the results of other investigators [5–7]. Enhanced oxidative stress and pro-inflammatory activity in adipose tissue are among potential mechanisms underlying decreased adiponectin synthesis in obesity [23, 24]. However, in the meta-analysis performed by Kuo and Halpern [25], no association of adiponectin serum levels with BMI in healthy, normal-weight to obese individuals was found. The authors suggest that the decreased adiponectin levels observed previously in the obese subjects may result from the co-existing obesity-related complications, including insulin resistance and cardiovascular diseases. In contrast, we did not observe any significant differences in the mean serum adiponectin level between the “healthy” obese individuals and these suffering from one or more obesity-related complications. Moreover, we did not find correlations between adiponectin serum concentration and basic biochemical and clinical parameters in obese individuals, even though in the literature such correlations were described [26, 27].

Resistin serum levels in our obese study participants were significantly higher than in normal-weight controls and correlated positively with triglycerides and negatively with HDL levels, which is also consistent with previous findings [28] and, as indicated by other authors, is a consequence of resistin-induced insulin resistance [13]. However, there are authors who observed an opposite correlations between resistin and lipoproteins concentrations [29]. Serum resistin levels were also higher in obese study participants who suffered from hypertension and/or met the diagnostic criteria for the metabolic syndrome. These findings are in agreement with the experimental data and clinical studies, were resistin was found to induce hypertension and insulin resistance via toll-like receptor 4 (TLR4) signaling [30].

Subsequently, the adiponectin–resistin (AR) index, proposed as an indicator of the metabolic risk in obesity, was significantly higher in serum of obese individuals, especially in these with MS, which again is consistent with previous findings [15].

Next, we investigated the adiponectin/resistin balance in adipose tissues and made an attempt to determine which adipose tissue depot plays a dominant role in synthesis of these two adipokines. To do this, we measured expression of *ADIPOQ* and *RETN* on the mRNA and protein levels. For both adipokines we noticed lack of correlation between the mRNA and protein concentrations, suggesting that posttranscriptional mechanisms may play a role in the regulation of expression of these two proteins [31]. We applied immunoassay method to measure adiponectin and resistin protein concentrations directly in protein extracts of the adipose tissues [17]. We showed that in the obese, adiponectin level is significantly decreased while resistin level is increased in SAT depot than in SAT of normal-weight individuals. This finding supports the theory of the dominant role of SAT in secretion of adipokines and inflammatory mediators and, therefore, in development of obesity-related complications [17, 32]. Moreover, in our group of obese individuals adiponectin serum concentrations correlated positively with *ADIPOQ* mRNA levels in SAT, as it was previously noticed by other authors [18, 19, 33, 34]. However, we cannot conclude that SAT is a dominant source of adiponectin or resistin in the organism, since we did not find correlations between these two adipokines’ protein levels in SAT and in serum regardless of body mass. On the other hand, we observed a negative correlation between the adiponectin serum concentration and protein level in VAT of obese individuals, that indirectly points to SAT as a main source of this adipokine in obesity. Nevertheless, defining whether VAT or SAT of the obese is more important in determination of adiponectin and resistin serum levels is difficult due to the factors interfering with adipokines release from the adipose tissue such as changes of protein structure, multimerization and local changes in inflammatory milieu [31].

Notably, the AR index calculated in adipose tissues was lower in SAT of obese individuals compared to the normal-weight controls, which is opposite to this seen in serum and did not reflect the metabolic risk in obese subject. The possible explanation of finding is that adipose tissue is not the only source of resistin, therefore its serum levels are higher than those measured in adipose tissue. This hypothesis is consistent with observation that indeed, resistin is produced by tissues other that adipose tissue, including e.g. epithelium and skeletal muscles [8].

Additionally, in obesity we observed a negative correlations between the adiponectin protein level in SAT and (i) adipose tissue content and (ii) triglyceride serum level, and a positive correlation with HDL serum concentrations. In case of resistin, we found a positive correlation between its protein level in VAT and low density lipoprotein (LDL) serum level in obese subjects. This is in line with previous data describing involvement of these adipokines in human pathology [3, 7, 14, 31, 35, 36].

Possible limitations of this study include a relatively small number of studied samples [37], although it exceeds the number of samples analyzed in previous studies regarding the expression of adiponectin [20, 32–35] and resistin [18, 19, 32] in human adipose tissue. In addition, in order to assure homogeneity of the studied group, we investigated only patients with class III obesity, while analysis of A/R balance in groups of obese patients with lower BMI could provide additional information regarding evolution of this parameter in subsequent classes of obesity.

## Conclusions

In conclusion, we studied the adiponectin/resistin balance in serum and in different adipose tissue depots in the obese and normal-weight subjects. We found that *ADIPOQ* and *RETN* mRNA levels did not correlate with adiponectin and resistin protein concentrations in adipose tissues originating from both obese and normal-weight subjects, suggesting a role for posttranscriptional mechanisms in the regulation of the amount of these two proteins. We also showed that obesity affects synthesis of adiponectin and resistin mainly in SAT. Finally, we found that the AR index based on measurements performed in the adipose tissues has a different prognostic value compared to the AR index in serum and does not reflect a metabolic risk in obese individuals.

## Abbreviations

*ADIPQ*: gene encoding adiponectin
AR: adiponectin–resistin index
BMI: body mass index
CRP: C-reactive protein
HDL: high density lipoprotein
IDF: International Diabetes Federation
LDL: low density lipoprotein
MS: metabolic syndrome
*RETN*: gene encoding resistin
SAT: subcutaneous adipose tissue
T2DM: type 2 diabetes
VAT: visceral adipose tissue
